# Analysis of real-world health care costs among immunocompetent patients aged 50 years or older with herpes zoster in the United States

**DOI:** 10.1080/21645515.2017.1324373

**Published:** 2017-06-12

**Authors:** Juliana L. Meyers, Shweta Madhwani, Debora Rausch, Sean D. Candrilli, Girishanthy Krishnarajah, Songkai Yan

**Affiliations:** a RTI Health Solutions, Research Triangle Park, NC, USA; b GSK, Philadelphia, PA, USA

**Keywords:** costs, herpes zoster, postherpetic neuralgia, managed care, retrospective claims analysis

## Abstract

Few peer-reviewed publications present real-world United States (US) data describing resource utilization and costs associated with herpes zoster (HZ) and postherpetic neuralgia (PHN). The primary objective of this analysis (GSK study identifier: HO-14–14270) was to assess direct costs associated with HZ and PHN in the US using a retrospective managed care insurance claims database. Patients ≥ 50 y at HZ diagnosis were selected. Patients were excluded if they were immunocompromised before diagnosis or received an HZ vaccine at any time. A subsample of patients with PHN was identified. Each patient with HZ was matched to ≤ 4 controls without HZ based on age, sex, and health plan enrollment. Incremental differences in mean HZ-related costs (“incremental costs”) were assessed overall and stratified by age. Multivariable regression models controlled for the effect of demographic characteristics, prediagnosis costs, and comorbidity burden on costs using a recycled predictions approach. Overall, 142,519 patients with HZ (9,470 patients [6.6%] had PHN) and 357,907 matched controls without HZ were identified. Resource utilization was greater among patients with HZ than controls. After adjusting for demographic and clinical characteristics, annual incremental health care costs for HZ patients vs. controls were $1,210 for patients aged 50–59 years, $1,629 for those 60–64 years, $1,876 for those 65–69 years, $2,643 for those 70–79 years, and $3,804 for those 80+ years; adjusted annual incremental costs among PHN patients vs. controls were $4,670 for patients 50–59 years, $6,133 for those 60–64 years, $6,451 for those 65–69 years, $8,548 for those 70–79 years, and $11,147 for those 80+ years. HZ is associated with a significant cost burden, which increases with advancing patient age. Vaccination may reduce costs associated with HZ through case avoidance.

## Introduction

Herpes zoster (HZ) is a cutaneous disease caused by the reactivation of latent varicella zoster virus from dorsal root or cranial nerve ganglia, which is present after primary infection with varicella.^1^ HZ is widely prevalent: nearly 1 in 3 individuals will develop HZ in their lifetime, accounting for 1 million cases each year in the United States (US).^2,3^ The risk of HZ increases sharply with age, affecting up to half of all people who live to 85 y of age and causing long-term morbidity.^1^ Since the early 1990s, the incidence of HZ in the US had been increasing gradually across age groups;^4-6^ and epidemiology continues to be monitored closely.

The Advisory Committee on Immunization Practices (ACIP) recommends HZ vaccination for individuals aged 60 y and older in the US.^7^ Efficacy rates in the pivotal trial of a live attenuated HZ vaccine (zoster vaccine live), the only HZ vaccine currently licensed in the US, were 51.3% among those aged 60 y and older and 37.6% among those aged 70 years and older.^8^ Clinical efficacy rate with an investigational HZ subunit vaccine (HZ/su) has been observed to be 89.8% among those aged 70 y and older, and was found to be similar among those aged 70 to 79 y (90.0%) and those aged 80 y and older (89.1%).^9^

HZ is typically characterized by the development of a rash and acute pain. Chronic pain, also known as postherpetic neuralgia (PHN), is the most commonly reported complication of HZ.^1,10^ PHN has been defined as pain persisting 1 to 6 months after either rash onset or healing. It has been estimated that approximately 18% of adult patients with HZ and 33% of HZ patients aged 79 y and older will develop PHN following an HZ episode.^11^ Antiviral therapy (using one of the 3 approved antiviral drugs in the US: acyclovir, valacyclovir, or famciclovir) with concurrent corticosteroid treatment is recommended for patients with HZ.^1^ Treatment options for PHN often consists of analgesics or opioids, tricyclic antidepressants, or gabapentin;^12-14^ however, these options are often not successful in addressing the pain of PHN.

There is evidence that HZ and PHN significantly affect health care resource utilization and medical costs, both in the US^15-20^ and in other regions.^10,21-23^ The cost of medical care for incident HZ cases in the US (based on the results of a study in Olmsted County, Minnesota) has been estimated at $1.1 billion annually.^15^ Dworkin and colleagues^16^ performed a retrospective database analysis to examine the costs associated with HZ and PHN in commercially insured, Medicaid, and Medicare populations in the US. Annual Medicare costs for patients with PHN were approximately double those of patients with HZ. Additionally, commercially insured and Medicaid patients with PHN had health care costs that were approximately 4 and 7 times more per year compared with patients with HZ, respectively. Another study reported that, among all patients regardless of age who had HZ, the annual per-patient HZ-related health care costs were $1,052, with substantially greater HZ-related costs observed among patients who developed PHN ($3,815).^17^

Despite published data documenting the increased risk of HZ associated with advancing age (e.g., ref. 2), there are limited recent real-world data describing health care resource utilization and costs related to HZ and PHN among patients aged 50 y and older. Given such limited data in the public domain, the primary objective of this study was to assess real-world costs of HZ and PHN among a US population of immunocompetent patients aged 50 y and older using data from a retrospective managed care insurance claims database, with results stratified by age group (e.g., 50 to 59 years; 80+ years). This analysis will provide context for the pharmacoeconomic evaluation of existing and emerging HZ vaccination strategies.

## Results

### Study population

The study population included 142,519 patients with HZ and 357,907 matched controls. From the HZ cohort, 9,470 patients (i.e., 6.6% of the final study sample) were identified as having PHN. Table 1 presents demographic characteristics of all study cohorts.

**Table 1.** Demographic characteristics and comorbidities for all study cohorts.

	HZ Cohort		Matched Non-HZ Cohort		PHN Cohort		Matched Non-HZ Cohort	
N	142,519		357,907		9,470		23,303	
Age at HZ index date (n, column %)								
50–59 years	67,573	47.41%	173,923	48.59%	3,042	32.12%	7,790	33.43%
60–64 years	30,778	21.60%	77,303	21.60%	1,603	16.93%	3,917	16.81%
65–69 years	9,436	6.62%	22,743	6.35%	757	7.99%	1,711	7.34%
70–79 years	19,161	13.44%	45,158	12.62%	1,949	20.58%	4,572	19.62%
80+ years	15,571	10.93%	38,780	10.84%	2,119	22.38%	5,313	22.80%
Mean (SD)	63.06	(10.68)	62.93	(10.66)	68.02	(12.19)	67.96	(12.35)
Median	60		60		65		64	
Range (minimum, maximum)	50	107	50	107	50	101	50	102
Sex (n, column %)								
Male	53,389	37.46%	134,672	37.63%	2,937	31.01%	7,014	30.10%
Female	89,130	62.54%	223,235	62.37%	6,533	68.99%	16,289	69.90%
Region (n, column %)								
Northeast	23,120	16.22%	64,620	18.05%	1,383	14.60%	4,161	17.86%
North Central	41,648	29.22%	99,419	27.78%	2,901	30.63%	6,791	29.14%
South	48,488	34.02%	115,116	32.16%	3,069	32.41%	7,134	30.61%
West	27,736	19.46%	75,443	21.08%	2,017	21.30%	5,043	21.64%
Missing/unknown	1,527	1.07%	3,309	0.92%	100	1.06%	174	0.75%
Payer type (n, column %)								
Commercial	98,897	69.39%	237,264	66.29%	4,635	48.94%	10,982	47.13%
Medicare	43,622	30.61%	120,643	33.71%	4,835	51.06%	12,321	52.87%
Health plan type (n, column %)								
Health maintenance organization	18,613	13.06%	48,491	13.55%	1,309	13.82%	3,194	13.71%
Preferred provider organization	76,107	53.40%	188,712	52.73%	4,531	47.85%	10,953	47.00%
Point of service	10,096	7.08%	26,267	7.34%	539	5.69%	1,359	5.83%
Other	28,716	20.15%	71,332	19.93%	2,635	27.82%	6,610	28.37%
Missing/unknown	8,987	6.31%	23,105	6.46%	456	4.82%	1,187	5.09%
Charlson comorbidities (n, column %)								
Congestive heart failure	1,379	0.97%	2,291	0.64%	155	1.64%	165	0.71%
Myocardial infarction	4,016	2.82%	7,216	2.02%	521	5.50%	740	3.18%
Peripheral vascular disease	4,554	3.20%	8,905	2.49%	565	5.97%	896	3.84%
Cerebrovascular disease	5,003	3.51%	9,416	2.63%	564	5.96%	920	3.95%
Dementia	846	0.59%	1,879	0.52%	96	1.01%	249	1.07%
Chronic pulmonary disease	11,135	7.81%	19,505	5.45%	1,286	13.58%	1,492	6.40%
Connective tissue disease	527	0.37%	756	0.21%	60	0.63%	76	0.33%
Ulcer disease	473	0.33%	857	0.24%	52	0.55%	75	0.32%
Diabetes without end organ damage	20,205	14.18%	42,387	11.84%	1,743	18.41%	3,031	13.01%
Depression	6,809	4.78%	12,631	3.53%	834	8.81%	824	3.54%
Use of warfarin	3,896	2.73%	8,000	2.24%	505	5.33%	800	3.43%
Hypertension	44,771	31.41%	95,024	26.55%	3,891	41.09%	6,937	29.77%
Hemiplegia	292	0.20%	536	0.15%	35	0.37%	45	0.19%
Moderate or severe renal disease	3,064	2.15%	5,370	1.50%	382	4.03%	468	2.01%
Diabetes with end organ damage	3,675	2.58%	6,954	1.94%	379	4.00%	580	2.49%
Any tumor^a^	142	0.10%	235	0.07%	9	0.10%	13	0.06%
Skin ulcers/cellulitis	5,963	4.18%	8,425	2.35%	593	6.26%	700	3.00%
Mild liver disease	1,969	1.38%	3,365	0.94%	174	1.84%	203	0.87%
Moderate or severe liver disease	114	0.08%	167	0.05%	15	0.16%	13	0.06%
Metastatic cancer	0	0.00%	0	0.00%	0	0.00%	0	0.00%
AIDS	0	0.00%	0	0.00%	0	0.00%	0	0.00%
Charlson Comorbidities Index score								
Mean (SD)	0.9	(1.26)	0.7	(1.10)	1.36	(1.51)	0.84	(1.22)
Median	0		0		1		0	
Range (minimum, maximum)	0	13	0	16	0	13	0	11
Baseline costs								
Mean (SD)	$2,037	($7,979)	$1,593	($7,037)	$3,465	($11,924)	$1,811	($7,018)
Median	$565		$377		$1,159		$476	
Range (minimum, maximum)	$0	$497,483	$0	$905,694	$0	$497,484	$0	$301,845

N = number of patients; n = number of patients in each category.

AIDS = acquired immunodeficiency syndrome; HZ = herpes zoster; ICD-9-CM = International Classification of Diseases, Ninth Revision, Clinical Modification; PHN = postherpetic neuralgia; SD = standard deviation.

Note: Percentages may not sum to 100% due to rounding.

a Includes ICD-9-CM code 172.xx malignant melanoma of skin, which was not considered an immune-compromised condition.

The mean (standard deviation [SD]) age among patients in the HZ cohort was 63.1 (10.7) years, and approximately two-thirds of patients were female. Patients were located in all 4 geographic regions across the US, with the largest percentages of patients residing in the South and North Central regions. Consistent with the distribution of patient age, approximately two-thirds of patients were covered by a commercial payer and one-third of patients were covered by Medicare. Over half of patients were enrolled in a preferred provider organization health plan. The mean (SD) Charlson Comorbidity Index (CCI) score was 0.9 (1.3) among patients in the HZ cohort, with the most common comorbidities observed being hypertension (31.4% of patients) and diabetes without end organ damage (14.2%). Demographic characteristics for the matched non-HZ cohort were generally similar to the HZ cohort; however, the mean (SD) CCI score for the matched non-HZ cohort was 0.7 (1.1).

The subgroup of patients in the PHN cohort were slightly older than patients in the HZ cohort (mean [SD], 68.0 [12.2] years) and included a slightly greater percentage of female patients relative to the HZ cohort. The geographic distribution and health plan types for patients in the PHN cohort were similar to the HZ cohort. Consistent with the older cohort age, the PHN cohort included a greater percentage of patients covered by Medicare compared with the HZ cohort. The matched non-HZ cohort was similar to the PHN cohort in terms of age, sex, geographic region, payer type, and plan type. Patients in the PHN cohort had a greater CCI score compared with patients in the matched non-HZ cohort (i.e., mean [SD], 1.4 [1.5] and 0.8 [1.2], respectively).

### Resource utilization

In the 1 y following the HZ index date (i.e., date of the first observed HZ diagnosis), 11% of patients in the HZ cohort had an inpatient visit compared with 8% of matched non-HZ controls, and 21% of patients in the HZ cohort had an emergency department (ED) visit compared with 13% of controls (data not shown). Approximately 64% of patients in the HZ cohort had an outpatient hospital visit compared with 53% in matched non-HZ controls. Nearly all patients in the HZ cohort had a physician office visit (98%) compared with 85% of controls (mean [SD] number of claims for office visits: 22.4 [27.2] among HZ patients and 16.6 [24.6] among controls). Almost 80% of HZ patients had a pharmacy claim compared with 69% of matched non-HZ controls, with HZ patients having a mean (SD) 20.6 (23.9) prescription claims versus 16.1 (21.9) prescription claims for matched non-HZ controls (data not shown). Table 2 presents the incremental health care resource utilization for patients with HZ vs. matched controls during the 1 y follow-up period, by age. In the 1 y following the HZ index date, HZ was associated with increases in inpatient and outpatient hospital visit claims, ED visit claims, physician office visit claims, and pharmacy claims; with the exception of pharmacy claims, all types of resource utilization tended to increase across age groups.

**Table 2.** Incremental health care resource utilization for patients with HZ versus matched controls during the 1-year follow-up period, by age.

	Inpatient Claims^a^		ED Claims		Outpatient Hospital Claims		Office Claims		Other Outpatient Claims		Pharmacy Claims	
Age	Mean	SE	Mean	SE	Mean	SE	Mean	SE	Mean	SE	Mean	SE
50–59 years	0.03	0.001	0.29	0.015	2.31	0.086	5.68	0.115	1.01	0.069	4.54	0.089
60–64 years	0.03	0.003	0.23	0.031	2.28	0.137	5.41	0.177	1.04	0.146	4.01	0.145
65–69 years	0.04	0.005	0.32	0.035	2.25	0.258	5.32	0.305	1.72	0.343	3.87	0.271
70–79 years	0.07	0.004	0.33	0.028	3.71	0.232	6.04	0.219	3.15	0.282	4.32	0.212
80+ years	0.15	0.006	0.54	0.037	4.20	0.217	6.68	0.227	6.52	0.363	4.45	0.277
Overall	0.05	0.001	0.31	0.011	2.72	0.066	5.79	0.080	1.99	0.075	4.45	0.070

ED = emergency department; HZ = herpes zoster; SE = standard error.

a Inpatient data represent one claim per visit.

### Costs

#### Unadjusted costs

**Figure 1.**
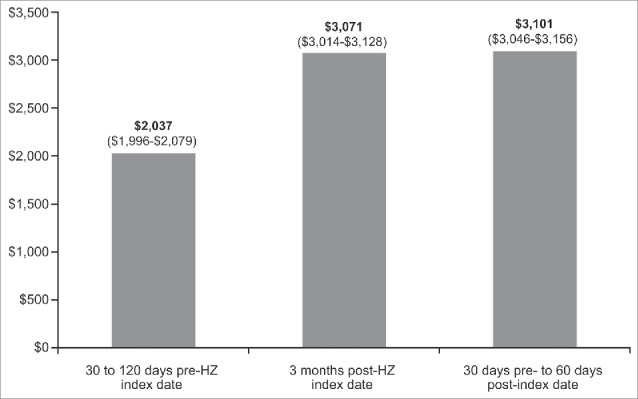
Total unadjusted all-cause health care costs (95% Confidence Intervals) by time period, All HZ patients. HZ = herpes zoster.

In the 30 to 120 d pre–HZ index date, unadjusted mean total health care costs from the pre-/post-analysis for all patients with HZ were $2,037 (Fig. 1). Total mean health care costs increased by $1,034 in the 3 months post–HZ index date, from $2,037 to $3,071, and by $1,064 in the 30 d pre–HZ index date to 60 d post–HZ index date, from $2,037 to $3,101.

Table 3 presents the unadjusted annual incremental difference in mean health care costs (“incremental costs”) by age and cohort. Patients with HZ and patients with HZ without PHN, respectively, accrued $2,564 and $2,023 more in annual mean health care costs vs. matched controls. Correspondingly, patients with PHN accrued $10,148 more in annual mean health care costs vs. matched controls, which was approximately 5 times greater than the annual incremental costs accrued by patients with HZ without PHN. Furthermore, incremental health care costs associated with HZ increased with advancing age, from $1,952 among patients aged 50 to 59 y to $5,442 among patients aged 80 years or older. Annual incremental health care costs also tended to increase with advancing age in the HZ non-PHN cohort. On the other hand, annual incremental health care costs were observed to be fairly consistent across all patient age groups for the PHN cohort.

**Table 3.** Unadjusted annual incremental health care costs by age and cohort.

	HZ Cohort vs. Matched Non-HZ Controls			HZ Non-PHN Cohort vs. Matched Non-HZ Controls			PHN Cohort vs. Matched Non-HZ Controls		
Age Group	Mean	Lower 95% CI	Upper 95% CI	Mean	Lower 95% CI	Upper 95% CI	Mean	Lower 95% CI	Upper 95% CI
Overall	$2,564	$2,417	$2,711	$2,023	$1,873	$2,173	$10,148	$9,477	$10,818
50–59 years	$1,952	$1,780	$2,123	$1,563	$1,389	$1,737	$10,193	$9,254	$11,131
60–64 years	$1,995	$1,647	$2,343	$1,524	$1,175	$1,872	$10,576	$8,516	$12,636
65–69 years	$2,166	$1,578	$2,754	$1,373	$764	$1,982	$11,290	$9,112	$13,467
70–79 years	$3,097	$2,629	$3,565	$2,424	$1,930	$2,918	$9,036	$7,579	$10,493
80+ years	$5,442	$4,879	$6,005	$4,688	$4,082	$5,294	$10,231	$8,720	$11,743

CI = confidence interval; HZ = herpes zoster; PHN = postherpetic neuralgia.

Table 4 presents the unadjusted incremental total all-cause health care costs in the first, second, and third months and in the first and second quarters, and 1 y following the HZ index date. Incremental costs associated with HZ were greatest in the first month after the HZ index date ($898) and decreased in the second month ($241) and third month ($192). Incremental costs associated with HZ were lower in the second quarter ($597) than in the first quarter ($1,330). This trend was also observed for HZ non-PHN patients, with incremental costs in the first month of $806, in the second month of $193, and in the third month of $151. Among PHN patients, incremental costs in month 1 were $2,180, which were approximately 2.7 times greater than the incremental costs in month 1 for HZ non-PHN patients. Incremental costs decreased in the second month post-HZ index date for PHN patients (to $914); however the decrease in incremental costs observed for PHN patients between months 1 and 2 (i.e., incremental costs decreased by approximately 58% between month 1 and 2 for PHN patients) was much lower than the decrease in incremental costs observed for HZ non-PHN patients between months 1 and 2 (i.e., incremental costs decreased by approximately 76% between month 1 and 2 for HZ non-PHN patients). Furthermore, PHN patients continued to accrue substantial incremental costs in quarter 2 post-HZ index date ($2,483), while the incremental costs for HZ non-PHN patients in quarter 2 post-HZ index date were much smaller ($462).

**Table 4.** Unadjusted incremental total all-cause health care costs by time period and cohort.

	HZ Cohort vs. Matched Non-HZ Controls			HZ Non-PHN Cohort vs. Matched Non-HZ Controls			PHN Cohort vs. Matched Non-HZ Controls		
Time Period	Mean	Lower 95% CI	Upper 95% CI	Mean	Lower 95% CI	Upper 95% CI	Mean	Lower 95% CI	Upper 95% CI
Month 1	$898	$867	$928	$806	$775	$838	$2,180	$2,046	$2,315
Month 2	$241	$216	$266	$193	$167	$219	$914	$797	$1,031
Month 3	$192	$168	$216	$151	$126	$176	$762	$658	$866
Quarter 1	$1,330	$1,278	$1,383	$1,150	$1,096	$1,204	$3,857	$3,624	$4,089
Quarter 2	$597	$542	$652	$462	$406	$519	$2,483	$2,233	$2,732
Year 1	$2,564	$2,417	$2,711	$2,023	$1,873	$2,173	$10,148	$9,477	$10,818

CI = confidence interval; HZ = herpes zoster; PHN = postherpetic neuralgia.

#### Adjusted costs

**Figure 2.**
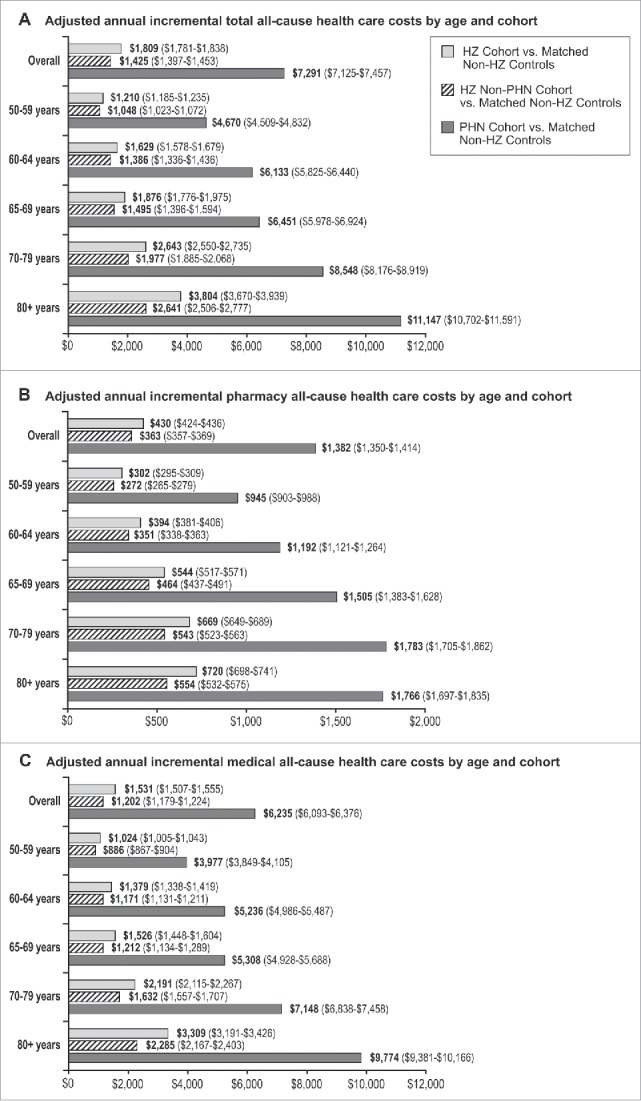
Adjusted annual all-cause health care costs (95% Confidence Intervals) by age and cohort, including (A) total costs, separated into (B) Pharmacy Costs,^a^ and (C) Medical Costs^a^. HZ = herpes zoster; PHN = postherpetic neuralgia. ^a^These data are from a post hoc analysis.

After adjusting for patients' demographic and clinical characteristics, the incremental costs associated with HZ were $1,809 (Fig. 2A). Correspondingly, the incremental costs associated with HZ without PHN were $1,425, and the incremental costs associated with PHN were $7,291 (i.e., the incremental costs associated with PHN were approximately 5.1 times greater than the incremental costs associated with HZ without PHN). Consistent with the unadjusted annual incremental costs, we found that adjusted annual incremental health care costs associated with HZ increased with advancing age: $1,210 among patients aged 50 to 59 years, $1,629 for those aged 60 to 64 years, $1,876 for those aged 65 to 69 years, $2,643 for those aged 70 to 79 years, and $3,804 among patients aged 80 y or older. This trend was also observed in patients with HZ without PHN ($1,048 among patients aged 50 to 59 years, $1,386 for those aged 60 to 64 years, $1,495 for those aged 65 to 69 years, $1,977 for those aged 70 to 79 years, and $2,641 among patients aged 80 y or older) and among patients with PHN ($4,670 among patients aged 50 to 59 years, $6,133 for those aged 60 to 64 years, $6,451 for those aged 65 to 69 years, $8,548 for those aged 70 to 79 years, and $11,147 among patients aged 80 y or older).

Fig. 2B presents the adjusted annual incremental pharmacy costs by age and cohort, and Fig. 2C presents the adjusted annual incremental medical costs by age and cohort (both estimated in a post hoc analysis). Overall, the adjusted annual incremental pharmacy costs were $430 for patients with HZ, $363 for patients with HZ without PHN, and $1,382 for patients with PHN. The adjusted annual incremental medical costs were $1,531 for patients with HZ, $1,202 for patients with HZ without PHN, and $6,235 for patients with PHN. Both incremental pharmacy and medical costs associated with HZ increased with increasing age: adjusted annual incremental pharmacy costs ranged from $302 among patients aged 50 to 59 y to $720 among patients aged 80 y or older; and adjusted annual incremental medical costs ranged from $1,024 among patients aged 50 to 59 y to $3,309 among patients aged 80 y or older. Correspondingly, both incremental pharmacy and medical costs associated with HZ without PHN and PHN also increased with increasing patient age.

**Figure 3.**
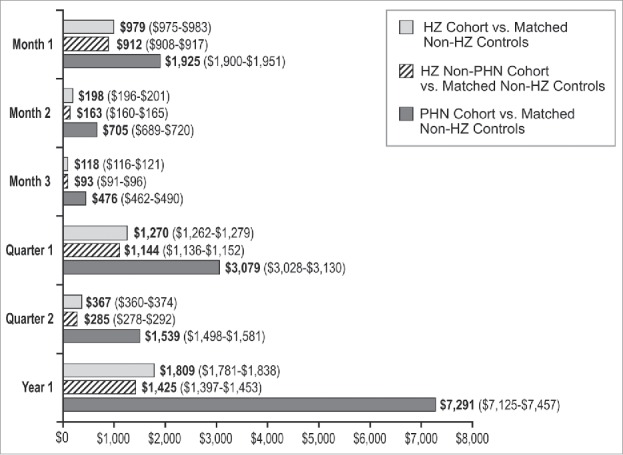
Adjusted incremental total all-cause health care costs (95% Confidence Intervals) by time period and cohort. HZ = herpes zoster; PHN = postherpetic neuralgia.

Figure 3 presents the adjusted incremental total all-cause health care costs by time period and cohort. Adjusted incremental costs associated with HZ were greatest in the first month after the HZ index date ($979) and decreased in the second month ($198) and third month ($118). Adjusted incremental costs associated with HZ were lower in the second quarter ($367) than in the first quarter ($1,270). This trend was also observed for HZ patients without PHN, with adjusted incremental costs in the first month of $912, in the second month of $163, and in the third month of $93. Among PHN patients, adjusted incremental costs in month 1 were $1,925, which were approximately 2.1 times greater than the adjusted incremental costs in month 1 for HZ non-PHN patients. Adjusted incremental costs decreased in the second month post-HZ index date for PHN patients (to $705), however the decrease in adjusted incremental costs observed for PHN patients between months 1 and 2 (i.e., adjusted incremental costs decreased by approximately 63% between month 1 and 2 for PHN patients) was much lower than the decrease in adjusted incremental costs observed for HZ non-PHN patients between months 1 and 2 (i.e., adjusted incremental costs decreased by approximately 82% between month 1 and 2 for HZ non-PHN patients). Furthermore, consistent with unadjusted results, PHN patients continued to accrue substantial adjusted incremental costs in quarter 2 post-HZ index date ($1,539), while the adjusted incremental costs for HZ non-PHN patients in quarter 2 post-HZ index date were much smaller ($285).

## Discussion

This retrospective database analysis was conducted to examine current incremental costs associated with HZ and PHN in a real-world population of immunocompetent patients. Patients with HZ were compared with an age- and sex-matched cohort of patients without HZ to assess the incremental costs associated with HZ in a demographically similar population. This study found that, on average, after adjusting for differences in characteristics of the underlying study populations, HZ was associated with an additional $1,809 in health care costs in the 12 months following the initial diagnosis, whereas PHN was associated with an additional $7,291 in health care costs during the same period. Additionally, the incremental costs associated with HZ were found to increase with increasing patient age.

Two previous analyses of the economic burden of HZ in the US have been conducted using the same database as was used in our analysis.^17,18^ Johnson and colleagues^18^ examined the economic burden of HZ among immunocompetent patients between January 1, 2007, and December 31, 2011. Similarly, White and colleagues^17^ examined health care resource utilization and cost burden among immunocompetent patients with HZ, as well as among patients with PHN, between 1998 and 2003. A slightly smaller proportion of patients in White and colleagues' study sample had PHN (4.4%, or 1,722 of the 39,209 patients with HZ who maintained health plan enrollment for 1 y following the index date) compared with our final study sample, of which 6.6% of patients had PHN.

Incremental costs associated with HZ were somewhat greater in our analysis compared with the results of previous analyses. White and colleagues^17^ observed that adjusted incremental annual health care costs associated with HZ among immunocompetent patients were $983 ($1,148 in 2013 US dollars), with costs generally increasing with increasing patient age from $1,020 ($1,191 in 2013 US dollars) among patients aged 50 to 59 y to $1,796 ($2,096 in 2013 US dollars) among patients aged 80 y or older. Additionally, Johnson and colleagues^18^ observed that incremental annual health care costs associated with HZ among all age groups of immunocompetent patients were $1,308 (reported in 2013 US dollars), with costs generally increasing with increasing patient age from $1,614 among patients aged 50 to 59 y to $2,256 among patients aged 80 y or older.

The differences in incremental costs reported between our study and the studies by White and colleagues^17^ and Johnson and colleagues^18^ may be due in part to differences in matching approaches. Specifically, in each of the other studies, HZ cases were matched to controls based on age and sex (as was done in our analysis), as well as pre–index date CCI score and pre–index date expenditures. In our study, to maximize the sample size, we matched on age and sex only, and then used multivariable models to control for other underlying characteristics, resulting in larger sample sizes than in the studies by White and colleagues and Johnson and colleagues. Because we did not match on pre–index date costs, it is possible that more patients with high costs during this period were included in our analysis than were found in the populations analyzed by White and colleagues^17^ and Johnson and colleagues.^18^

This study was conducted using an administrative claims database and has limitations common to all retrospective database analyses. Diagnoses were taken from administrative billing records, which may be subject to miscoding or undercoding, and no clinical data or electronic medical records were available to confirm diagnoses or clinical events. No ICD-9-CM (International Classification of Diseases, Ninth Edition, Clinical Modification) diagnosis code exists for PHN. Patients with PHN were identified using a published algorithm;^24^ however, patients may have been incorrectly identified as having PHN, or patients with PHN may not have been identified for inclusion. Patients with HZ and PHN who did not seek medical care were not included in the analysis; therefore, this study likely overestimated the costs per patient associated with HZ and PHN. Cost data were based on health plan–paid amounts and patient copayments, which reflects only the cost incurred to payers. Health care services or prescription medications paid for completely out of pocket or by other supplemental insurance were not observed; nor were any medications that may have been administered on experimental protocols. The study database did not include over-the-counter medications or other treatments not covered by insurance. Therefore, although this study captured the costs of HZ care to payers, the total costs associated with HZ management may be underestimated.

Patients were identified as having a new HZ episode based on a 6-month “clean” period during which the patient was enrolled in the health plan but did not have an HZ diagnosis during that period. Patients with HZ or PHN without continuous health plan enrollment during the 6-month clean period were not included in our study. Further, some patients may be incorrectly flagged as having a new HZ episode if they had no HZ-related medical claims during the 6-month clean period (i.e., had HZ diagnosed before the start of the study's data time period, but no claims in the first 6 months of the study data).

Pre-/post-analyses have several limitations inherent to the study design.^25^ Limitations specific to our analysis include time period bias and the lack of a control group, given that patients are compared with themselves before and after their HZ index date. In addition, costs reported in claims data are subject to a skewed distribution, although multivariable models were used for the statistical analyses in this study in an attempt to control for this effect. Further, there exists the potential for health care resource utilization and costs related to the workup of HZ (e.g., office visits, laboratory tests, but without an HZ diagnosis code) occurring before the initial HZ diagnosis, with such utilization not necessary for non-HZ controls. To account for this potential source of bias, the pre–HZ index date period was selected as 30 to 120 d before the first observed HZ diagnosis (e.g., to exclude the period when an HZ workup would likely occur). This study was designed to assess incremental costs and evaluated a specifically selected control population and results should not be extrapolated to the entire US population. Finally, this study examined patients in a commercial claims population. Although the data are representative of the US commercially insured population, results may be different for patients in fee-for-service Medicare or Medicaid programs.

Results of this study suggest that HZ is associated with significant cost burden, with health care costs of approximately $1,809 in patients aged 50 y or older covered by commercial insurance. The greatest cost burden was typically observed in the first month following the initial HZ diagnosis (e.g., the acute period), and the burden of HZ was found to increase with advancing patient age. Results of this analysis may be of interest to physicians and health care providers treating patients with HZ and to decision makers involved in the treatment and prevention of HZ. Case avoidance by way of effective vaccination strategies may reduce the economic burden of HZ. Previous studies have shown the cost-effectiveness of HZ vaccination to depend on age at vaccination, with greater cost-effectiveness observed among patients aged 60 to 70 y than among patients aged 50 to 59 y.^26-29^ These studies' findings are consistent with ACIP's current recommendation in favor of HZ vaccination for patients aged 60 y and older.^7^ As new HZ vaccination strategies emerge, our analysis provides up-to-date resource utilization and cost data to inform future pharmacoeconomic and cost-effectiveness analyses in populations that are vulnerable to HZ infection.

## Patients and methods

### Data source

This retrospective cohort analysis (GSK study identifier: HO-14–14270) used detailed resource utilization and cost data from the Truven Health Analytics *MarketScan* Commercial Claims and Encounters and the Medicare Supplemental and Coordination of Benefits administrative claims databases, composed of commercially insured persons enrolled in managed care health plans throughout the US. The Truven Health Analytics databases contain medical claims, pharmacy claims, and drug utilization data for nearly 40 million unique individuals.^30^ This study used the most recent 5 y of data available at the time the study was conducted (July 1, 2008, through June 30, 2013).

### Study population

**Figure 4.**
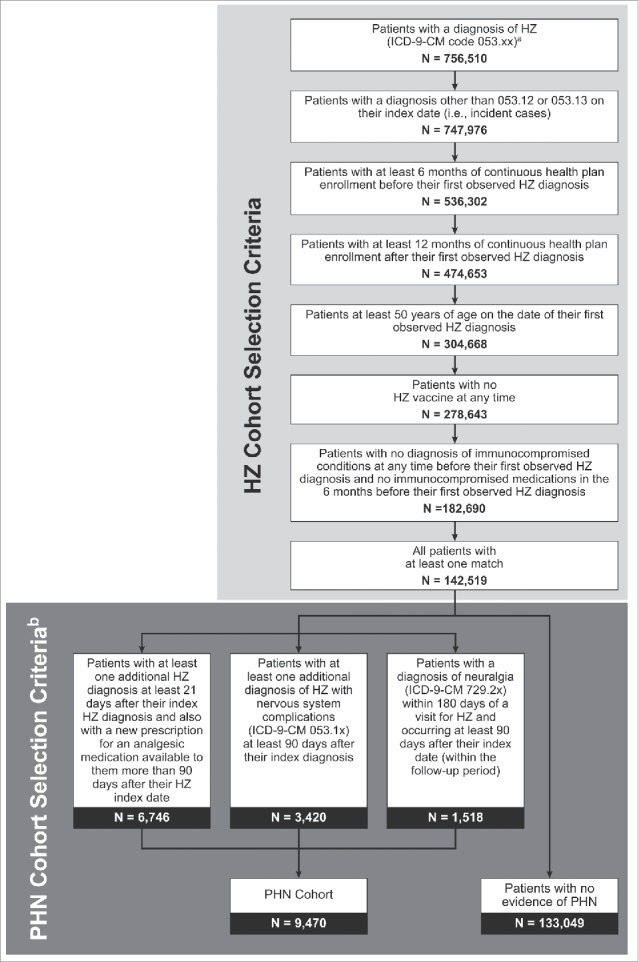
Sample selection flow chart: HZ and PHN Cohorts. HZ = herpes zoster; ICD-9-CM = International Classification of Diseases, Ninth Revision, Clinical Modification; PHN = postherpetic neuralgia; PPV = positive predictive value. ^a^Klompas and colleagues (2011)^24^ found that ICD-9-CM code 053.xx detected HZ with 98% sensitivity and 93% PPV. ^b^Klompas and colleagues (2011)^24^ found the highest sensitivity (86%) and PPV (78%) associated with the criteria proposed here to identify PHN patients. Additional criteria were also assessed by Klompas and colleagues, but the sensitivity and PPV were found to decrease with the additional criteria.

Patients with a diagnosis of HZ (ICD-9-CM diagnosis code 053.xx) were selected. The date of the first observed HZ diagnosis designated the index date. Patients were required to be aged 50 y or older at the index date and to have continuous health plan enrollment 6 months before the index date through 12 months after the index date. A cohort of patients with both HZ and PHN was identified from the overall HZ cohort. Because no ICD-9-CM diagnosis code exists for PHN, patients with PHN were identified using a published algorithm.^24^ Patients were excluded from both the HZ cohort and the PHN cohort if they had an immunocompromised diagnosis, received a medication indicating an immunocompromised condition, or received an HZ vaccine at any time before their index date. As we were interested only in new HZ episodes (and not continuation of care for ongoing episodes or patients with pre-existing PHN), patients were excluded from the HZ cohort if their first observed HZ diagnosis was ICD-9-CM diagnosis code 053.12 (postherpetic trigeminal neuralgia) or 053.13 (postherpetic polyneuropathy). Each HZ patient was matched to up to 4 patients without HZ (i.e., controls) based on age, sex, and health plan enrollment, with control patients assigned the index date of their HZ patient. For each control, the index date of the case was assigned (i.e., each control had the index date of their respective case). Fig. 4 presents the selection criteria for the HZ cohort and the PHN cohort.

This study was reviewed by the Office of Research Protection and Ethics at RTI International. As the study contained data that was retrospective, de-identified, and anonymous, RTI International's institutional review board committee determined that this study did not qualify as research with human subjects.

### Study outcomes

The primary end point for this analysis was total incremental difference in mean health care costs (“incremental costs”) associated with HZ and PHN. Total HZ-related incremental health care costs were assessed using pre-/post-analysis and matched comparison analysis. Total PHN-related incremental health care costs were assessed using a matched comparison analysis only. For the pre-/post-analysis, HZ-related incremental costs were estimated by subtracting mean all-cause health care costs accrued by patients in the 30 to 120 d before their HZ diagnosis from mean all-cause health care costs accrued by patients in the 3 months after their HZ diagnosis as well as in the 30 d before to 60 d after their HZ diagnosis. The period of 30 to 120 d before HZ diagnosis was selected to account for any HZ-related health care visits that may have occurred in the weeks leading up to the HZ diagnosis (i.e., HZ prodromal workup visits). The difference in mean costs for the 2 periods was the HZ-attributable incremental costs during the 3 months after HZ diagnosis.

The matched comparison analysis was conducted for the HZ and PHN cohorts to estimate HZ- and PHN-related incremental costs. HZ-related incremental costs were estimated as the difference between mean all-cause health care costs accrued by patients in the HZ cohort and mean all-cause health care costs accrued by patients in the matched non-HZ cohort. The difference in mean costs for the 2 cohorts was the HZ-attributable incremental costs. HZ-related incremental costs were estimated for months 1, 2, and 3 following HZ index date; quarter 1 (i.e., sum of months 1 through 3) and quarter 2 (i.e., sum of months 4 through 6) following HZ index date; and 1 y (i.e., sum of months 1 through 12) following HZ index date.

Similarly, for the HZ with PHN cohort, the PHN-related incremental costs were those attributable to PHN relative to matched non-HZ controls. PHN-related incremental costs were estimated as the difference between mean all-cause health care costs accrued by patients in the PHN cohort and mean all-cause health care costs accrued by patients in the matched non-HZ controls cohort. The difference in mean costs for the 2 cohorts defined the PHN vs. matched non-HZ-attributable incremental costs. Among all patients with PHN, PHN-related incremental costs were estimated for the same time periods used for the estimates of HZ-related incremental costs (months 1, 2, and 3; quarter 1 and 2; and 1 y following HZ index date).

Categories of health care utilization and costs that were evaluated and reported included inpatient stays, ED visits, office visits, hospital outpatient encounters, pharmacy prescriptions, other ancillary health care encounters, and total health care utilization. Using matched comparison analysis, HZ-related incremental health care resource utilization was estimated as the difference in mean number of claims for each health care resource utilization category between patients in the HZ cohort and patients in the matched non-HZ cohort. The difference between the 2 cohorts was the HZ-attributable resource utilization.

### Data analyses

All analyses were conducted using *SAS* version 9. Study outcomes were analyzed descriptively through the tabular display of mean values, medians, ranges, and SDs of continuous variables of interest and frequency distributions for the categorical variables. Costs were assessed from the perspective of the entire disease burden and included patient, health plan, and any coinsurance payments. All cost data were adjusted to 2013 US dollars using the Medical Care Component of the US Consumer Price Index.

Demographic characteristics and other patient-specific characteristics (e.g., age, sex, geographic location, payer type, and plan type) were collected at patients' index date, while CCI score was calculated over the 6-month pre–index date period. All-cause costs incurred during the 30 to 120 d before the HZ index date were calculated.

Health care utilization and costs were compared between the cases and matched controls using univariate tests (i.e., paired Student t-tests for continuous measures and McNemar chi-square test of association for categorical measures). The following group comparisons were undertaken: HZ cases vs. matched non-HZ controls; HZ non-PHN cases vs. matched non-HZ controls (this comparison was a post hoc analysis); and PHN cases vs. matched non-HZ controls. The outlined group comparisons were conducted overall and stratified by age groups (i.e., 50–59 years, 60–64 years, 65–69 years, 70–79 years, and 80+ years).

To assess differences in total health care costs for the matched comparison analysis, we used generalized estimating equations (GEEs) with a log-link function for the mean and a gamma distribution for the error term to resolve the issue of skewed cost distribution that is common in claims data.^31^ In the matched comparison analysis, regression models were estimated to determine adjusted incremental total HZ-related costs following the HZ index date. One regression model was run that took into account all monthly periods of interest following the HZ index date (i.e., month 1, month 2, month 3). Another regression model was run that took into account the 2 quarterly periods of interest following the HZ index date (i.e., quarter 1 and quarter 2), and a third regression model was run for the 1 y period following the HZ index date. The regressions for monthly costs and for quarterly costs were GEE models with a log link for the mean and a gamma distribution for residuals, adjusting for the correlation within patient observations by allowing the GEE to determine the correlation structure present in the data in an iterative process. The regression for 1-year costs was also a GEE model with a log link for the mean and a gamma distribution for residuals. All models took the following general form:

$$\text{Log}\left(\text{E}\left(\text{Y}\right)\right){\text{ = β}}_{\text{0}}{\text{+ β}}_{\text{1}}{\text{X}}_{\text{i}}{\text{+ β}}_{\text{2}}{\text{CLIN}}_{\text{i}}{\text{+ β}}_{\text{3}}{\text{COHORT}\;+\text{ β}}_{\text{4}}{\text{AGE}}_{\text{i}}{\text{+ β}}_{\text{5}}{\text{COSTS}}_{\text{i}}{\text{+ β}}_{\text{6}}{\text{PERIOD}}_{\text{i}}\text{,}$$

where Y was the total health care costs in the period, βs were coefficients to be estimated, X_i_ was a vector of background characteristics (i.e., sex, geographic region, health plan type, payer type); CLIN_i_ was a vector of clinical characteristics such as CCI score during the pre–index date period; COHORT was a measure indicating whether an observation represented the HZ, PHN, or non-HZ cohort; AGE_i_ was a vector of binary variables for age categories (i.e., 50–59 years, 60–64 years, 65–69 years, 70–79 years, 80+ years); COSTS_i_ was a vector of flags for the 5% percentiles of the costs in the 30 to 120 d before the HZ index date; and PERIOD_i_ was a class variable for period included in the monthly and quarterly GEE models only. Additionally, for the monthly and quarterly GEE models, interaction terms for the cohort and period were included to account for the effect of changes in costs over time.

A recycled predictions approach was used to assess differences in costs between cases and controls by predicting costs for all patients according to HZ status (i.e., cases and controls), using each patients' own values.^32^ Specifically, for the recycled predictions approach, following model estimation, all patients' data were input back into the model twice, the first time setting the HZ variable equal to 1 for all patients (i.e., assume all patients have HZ, regardless of whether or not they actually had HZ) and the second time setting the HZ variable equal to 0 for all patients (i.e., assume no patients have HZ, regardless of whether or not they actually had HZ). The mean predicted value assuming all patients had HZ and the mean predicted value assuming all patients did not have HZ were reported. This process was repeated for each time period (i.e., month 1, month 2, month 3, quarter 1, and quarter 2 post–index date). Additionally, adjusted results were reported for the following patient populations: HZ patients, matched controls, HZ patients with no PHN diagnosis, and PHN patients. When calculating adjusted results for the subpopulations of patients with HZ with no PHN diagnosis only the subpopulation of patients with HZ with no PHN diagnosis and their corresponding matched controls were evaluated. Results were reported overall and for each age category (i.e., 50–59 years, 60–64 years, 65–69 years, 70–79 years, 80+ years). Adjusted incremental HZ-related health care costs were reported as adjusted costs for the HZ cohort minus adjusted costs for the matched non-HZ comparison cohort.

## Abbreviations

AIDS: acquired immunodeficiency syndrome
CCI: Charlson Comorbidity Index
ED: emergency department
GEE: generalized estimating equation
HZ: herpes zoster
ICD-9-CM: International Classification of Diseases, Ninth Edition, Clinical Modification
PHN: postherpetic neuralgia
PPV: positive predictive value
SD: standard deviation
SE: standard error
US: United States
